# DIMINISHED COMPLEXITY OF RELIGIOUS BELIEF AMONG DROPOUTS FROM A LONGITUDINAL STUDY OF COPING

**DOI:** 10.1093/geroni/igz038.1714

**Published:** 2019-11-08

**Authors:** Jordan A Anderson, Shaelyn M Harris, Erica L Nelson, Ingrid Teuber, Andrew Futterman

**Affiliations:** Department of Psychology, Loyola University Maryland, Baltimore, Maryland, United States

## Abstract

Selective attrition is a common problem in longitudinal studies of older adults. Dropout is due to many factors, but frequently health concerns figure prominently as a reason for attrition. In light of previous work that suggests health problems reduce complexity of religious and other social involvements, the current study examines complexity of religiousness among dropouts and continuers in a longitudinal study of religion and health in later life. A random sample of 287 older adults living in Worcester, MA was assessed at two times of measurement 12 months apart using interview-based measures of religious orientation (Batson, Schoenrade, and Ventis, 1993) and health (OARS). Of the 287 who began the study, 72 dropped out and were not available to be assessed at 12 month assessment. Using Mplus, a three-factor model of Ends, Means, and Quest orientations demonstrated a good fit to the data in both dropout and continuer subsamples (e.g., CFI’s equaled .959 and .966, respectively). Diminished correlations between Ends, Means, and Quest orientations in the dropout vs. continuer subsample suggests diminished complexity of religious orientation among dropouts. Dropouts were more seriously ill, had higher levels of functional impairment, and demonstrated lower SES, suggesting increased vulnerability and fewer resources may have been the reason for dropping from the study as well as one possible cause of their diminished religious complexity.

